# Efficacy of Rose Stem Cell‐Derived Exosomes (RSCEs) in Skin Treatment: From Healing to Hyperpigmentation Management: Case Series and Review

**DOI:** 10.1111/jocd.16776

**Published:** 2025-01-15

**Authors:** Lidia Majewska, Karolina Dorosz, Jacek Kijowski

**Affiliations:** ^1^ ESME Clinic Kraków Poland; ^2^ University of Chicago Chicago Illinois USA; ^3^ Małopolska Centre of Biotechnology, Stem Cell Laboratory Jagiellonian University Kraków Poland

**Keywords:** Anti‐inflammatory effects of exosomes, Exosome‐driven therapies in aesthetics, Exosome innovations in scar repair, Exosomes in skin regeneration, Hyperpigmentation management using exosomes, Plant‐derived exosome therapies, Regenerative medicine with exosomes, Rose stem cell exosomes in dermatology, Skin barrier enhancement through exosomes, Wound healing with exosome‐based treatments

## Abstract

**Objective:**

To present and analyze eight clinical cases illustrating the use of rose stem cell‐derived exosomes (RSCEs) in treating various dermatological conditions and to review current literature on plant‐derived exosomes in medicine and dermatology.

**Background:**

RSCEs possess low cytotoxicity, high biocompatibility, and effective cellular uptake, making them promising agents for dermatological therapies. A literature review included in the introduction and discussion covers the broader role of plant‐derived exosomes, highlighting their therapeutic potential in skin treatment.

**Methods:**

A case‐by‐case analysis was conducted on eight patients with conditions including atopic dermatitis (AD), hyperpigmentation, scarring, wounds, melasma, and antiaging concerns. Each case provided insights into RSCEs' efficacy, with a focus on their antioxidant and anti‐inflammatory properties, as well as specific learning points derived from clinical observations.

**Results:**

The cases demonstrated RSCEs' multifaceted therapeutic effects across different skin conditions, supporting their role in enhancing skin regeneration, wound healing, and reducing hyperpigmentation and scarring. The literature review underscored RSCEs' unique bioactivity, suggesting mechanisms for their observed effects, including anti‐inflammatory and rejuvenating properties, which contributed to favorable clinical outcomes.

**Conclusion:**

RSCEs show potential as a valuable treatment in dermatology, as evidenced by the positive results across multiple skin conditions and their alignment with existing literature on plant‐derived exosomes. This case series emphasizes the need for further randomized and controlled clinical trials to confirm these preliminary findings and expand RSCEs' clinical application in dermatology.

## 1 Introduction

Extracellular vesicles (EVs) are a significant mediator of intercellular communication in both prokaryotic and eukaryotic organisms regulating many biological processes [1, 2]. They are spherical, double‐membrane structures ranging between 30 and 1000 nm in size [1, 2]. EVs contain a unique mixture of nucleic acids, proteins, and lipids reflective of their producer cells' characteristics. Consequently, EVs can be used as an effective therapeutic agent offering similar benefits to cell‐based therapies, while eliminating the possibility of autoimmune rejection [3].

Mesenchymal stem cell EVs (MSC EVs) have gathered particular interest due to their immunomodulatory and regenerative functions. Many studies show that MSC EVs exhibit anti‐inflammatory, antiaging, and wound healing stimulation effects across in vitro and in vivo models [4, 5, 6]. EVs cause these positive therapeutic results as they act as the main mediator of MSC‐secreted factors contributing to the paracrine effect [7]. Further, MSC EVs are considered safe to use as studies demonstrate that they do not exhibit oncogenic effects [6, 8]. Thus, MSC‐derived EVs show promising clinical potential to counteract many physiological conditions, including neurological, cardiovascular, autoimmune, renal, musculoskeletal, liver, respiratory, ocular, dermal, and cancer [6, 7, 8, 9].

Human adipose tissue stem cell (ACS) EVs remain a novelty within clinical use; however, they are already employed in treatment of dermal conditions [10, 11]. Animal studies show that ACS EVs induce epidermal barrier regeneration via an increase in the production of ceramides, dihydroceramides, sphingosine, and S1P [12]; reduce inflammation by lowering pro‐inflammatory cytokine level [13]; decrease TSLP level (itch‐inducing cytokine) [14]; stimulate human dermal fibroblasts to produce collagen and elastin [15].

Despite their promising effects, human ACS EV cosmetic products are not currently approved in the European Union [16]. However, an effective alternative substrate available on the market is plant‐derived EVs. Plants are capable of producing EVs under stimuli caused by bacteria or viruses [17, 18]. Plant‐derived EVs are biocompatible, have low immunogenicity, low toxicity, and can be engineered to target specific receptors [17]. Due to their small size, they can also penetrate the blood–brain barrier, making them a promising agent for treatment of neurological or oncological conditions [17].

Plant exosome‐like nanoparticles (PENs) represent a new opportunity and concept in the therapy of many diseases, and thus, their full therapeutic potential is not yet known. However, their effectiveness has been confirmed in numerous disease models [18, 19, 20, 21, 22, 23]. The diversity and availability of plants allow for the isolation of exosomes with therapeutic activity and broad applicability. Numerous studies document the effects of plant exosomes in the treatment of acute and chronic colitis as well as inflammatory bowel disease [19, 20, 22].

Another application of PENs is their use as drug delivery systems (DDS) to specific cells [22, 23]. PENs can target specific cells, tissues, or organs [21] especially since the commonly used synthetic carriers have low biocompatibility, short retention time higher toxicity, and poor targeting efficiency [24]. PENs guarantee high biocompatibility and stability under a variety of physiological conditions (e.g., different pH levels). PENs can also be used as vectors to carry siRNAs/miRNAs and chemotherapeutic drugs [18].

Due to the above, preclinical and clinical studies based on PENs have been registered, for example, grape exosome‐like nanoparticles (GELNs) (study NCT01668849) and ginger and aloe (study NCT03493984). GELNs have been used to manage oral mucositis pain induced by radiation and chemotherapy in patients with head and neck cancer. Ginger‐derived exosome‐like nanoparticles (GDENs) and aloe exosome‐like nanoparticles have been registered for studies in patients with polycystic ovary syndrome. However, none of these studies have yet been completed.

In 2023, Won et al. [25] demonstrated the positive impact of plant EVs on skin cell function such as fibroblasts' proliferation, stimulation of collagen production, reduction of melanin content in melanocytes, and inhibition of inflammation.

This publication documents as one of the first ones, the effectiveness of plant EVs in modulating skin functions. The cases presented in this publication are based solely on the use of plant‐based EVs (derived from Damask rose stem cells). Thus, this case series serves to offer new evidence for successful clinical use of plant‐derived EVs across various dermal conditions.

### 1.1 Product Specification

In each of the presented cases, the product ASCEplus Derma Signal Kit/SRLV, ExoCoBio Inc., Seoul, South Korea, containing 20 mg of lyophilized rose stem cell‐derived exosomes (RSCEs), was used. The product is packaged in two containers—one containing the lyophilized powder (with RSCEs constituting 40% of the composition) and the other containing 5 mL of solution/diluent used to dissolve the lyophilizate. The product is prepared ex tempore and requires storage at a temperature of 2°–8°C (Table 1). Table of contents of the lyophilized powder and the diluent are presented in the Supporting Information section.

**TABLE 1:** Dosage and potential skin applications of ASCEplus/SRLV.

Parameter	Details
Product name	ASCEplus Derma Signal Kit/SRLV, ExoCoBio Inc., Seoul, South Korea
Contents	20 mg lyophilized rose stem‐cell‐derived exosomes (RSCEs)
Packaging	Two containers: one with lyophilizate and one with 5 mL solution to dissolve the lyophilizate
Storage temperature	2°–8°C
Potential skin applications	– Treatment of atopic dermatitis
	– Stimulation of wound healing
	– Scar remodeling
	– Reduction of hyperpigmentation

## 2 Potential Applications of Rose Stem Cells Exosomes

A summary of the following cases along with learning points is presented in the table below (Table 2).

**TABLE 2:** Summary of potential applications of rose stem cell exosomes.

	Age	Gender	Presentation	Treatment	Results	Learning points
1.	21	F	Atopic dermatitis (AD)	ASCEplus/SRLV topically for 2 weeks.	Significant reduction in skin changes, decrease in eczema, and complete cessation of itching on the hand treated with ASCEplus/SRLV.	Efficacy of ASCEplus/SRLV tin reducing inflammation and promoting skin barrier repair, indicating its potential as a therapeutic option for AD management. Duration of effects‐he observed relief lasting several weeks highlights that exosomes could provide longer‐lasting benefits compared to conventional treatments. Mechanism of action in inflammatory conditions—exosomes contribute to immune modulation as evidenced by the reduction in AD flare‐ups. Patient tolerance and safety—the absence of adverse effects indicates a favorable safety profile, suggesting exosome‐based products may be well‐tolerated in patients with sensitive or compromised skin, like those with AD.
2.	36	F	Scar in the midface following autologous skin graft.	Combination therapy with ASCEplus/SRLV and two Dermapen microneedling sessions (0.5 mm) over 12 days.	Noticeable improvement of skin color, evening of skin surface sculpture, and reduced visibility of blood vessels. Pulling sensation around the nostril completely disappeared.	Combination therapy potential—the use of exosomes alongside microneedling offers synergistic effects, enhancing skin regeneration and scar remodeling. Role of exosomes in scar remodeling—acceleration of healing process and improving skin texture suggesting exosomes could be valuable in managing postoperative scars and other facial skin irregularities. Short‐term application efficacy—faster recovery protocol for facial scars. Patient tolerance and safety—the procedure was well‐tolerated indicating that treatment is a safe approach for facial scars, even on sensitive skin graft areas.
3.	33	F	Second‐degree burn in the area of the left corner of the mouth.	ASCEplus/SRLV applied twice daily to the burned skin surface (Days 2–6).	Complete healing on the fourth day of treatment.	Accelerated healing—significant shortening of the healing process in second‐degree facial burns. Prevention of hyperpigmentations. Reduced scarring and improved skin quality. Patient safety and comfort—reduction of the downtime.
4.	39	F	Second‐degree burn (sunburn) on the upper surface of the right forearm.	ASCEplus/SRLV applied twice daily to the burned skin surface (Days 3–10).	Complete healing on the seventh day of treatment.	Rapid healing despite blister disruption—complete healing within 7 days suggests exosomes may accelerate tissue repair, even when the skin barrier has been further compromised by blister removal. Prevention of post‐inflammatory hyperpigmentation—the absence of residual pigmentation indicates that exosome treatment could be effective in preventing hyperpigmentation, even in darker skin phenotypes prone to discoloration. Enhanced skin recovery—the treatment promoted not only faster healing but also improved post‐burn skin quality, which is essential for visible areas like the forearm. Patient safety and tolerance—treatment was well‐tolerated, highlighting exosomes as a potentially safe option for managing sunburn‐related injuries on more pigmented skin types.
5.	42	F	Lacerated wound on the left lower leg due to a bicycle accident.	ASCEplus/SRLV applied twice daily to the wound surface.	Accelerated wound closure in the fourth day of treatment.	Rapid wound closure shows exosomes' potential to expedite healing in slow‐healing injuries. Enhanced regenerative support—exosomes can still significantly impact tissue repair in delayed healing cases. Reduced risk of infection and complications due to faster wound closure. Positive patient tolerance—exosomes as safe option for managing trauma‐related injuries.
6.	37	M	Antiaging treatments—skin healing after ablative fractional laser treatment.	Combination therapy with ASCEplus/SRLV applied twice daily to the treated surface (one side of the neck) for 6 days following one session of Er:YAG laser.	Complete healing of the exosomes' treated side on the sixth day after the treatment.	Accelerated healing with exosomes—the exosome‐treated side healed completely in 6 days, while the untreated side was still visibly healing, suggesting that exosomes can significantly speed up recovery after laser treatments. Reduction in post‐treatment discomfort—the exosome‐treated side experienced less itching, dryness, and redness, indicating that exosomes may improve patient comfort and reduce post‐procedural irritation. Potential for enhanced skin quality—faster and more comfortable recovery with exosomes highlights their potential as a supportive treatment in antiaging protocols, improving both healing time and skin texture.
7.	61	F	Facial skin photoaging/hyperpigmentations	Combination therapy with ASCEplus/SRLV used for 5 days and one Dermapen microneedling session (depth 1–1.5 mm).	Noticeable skin brightening and reduction in pigmentation.	Visible reduction in hyperpigmentation suggesting exosomes' effectiveness in mature skin with accumulated sun damage. Improved skin hydration and quality—combined treatment (exosomes+ microneedling) may boost skin barrier function and moisture retention. Potential for non‐protected skin—positive outcome in a patient with no history of photoprotection highlight the potential of this treatment for skin repair in those with sun exposure history. Immediate aesthetic benefits—ideal for patients seeking rapid results.
8.	42	F	Melasma, particularly intense on the forehead and the cheeks.	Combination therapy with ASCEplus/SRLV used after treatment for 7 days and two Dermapen microneedling (depth 1–1.5 mm)sessions, 4 weeks apart.	Significant reduction of melasma, significantly improved skin texture.	Effective melasma reduction—even in severe cases of melasma in challenging areas like the forehead and the cheeks. Long‐lasting results—noticeable improvement maintained 12 weeks post‐treatment. Enhanced skin repair and regeneration—this combination treatment improves melanin level regulation and helps prevent recurrence of pigmentation. High patient tolerance and compliance—the spaced treatment protocol with weekly exosome application is well‐tolerated and manageable for patients.

### 2.1 Atopic Dermatitis

A 21‐year‐old woman had been treated for atopic dermatitis (AD) since she was 3 years old. Clinically, she experienced lichenified eczema with significant changes on the backs of both hands and minor changes on the face. Additionally, the changes on her hands were accompanied by itching, which intensified under stress. During childhood, she was treated with topical glucocorticosteroids in periods of exacerbation and itching. After puberty, the changes decreased, reoccurring periodically—then she used calcineurin inhibitors. Additionally, emollients were applied daily.

**FIGURE 1:**
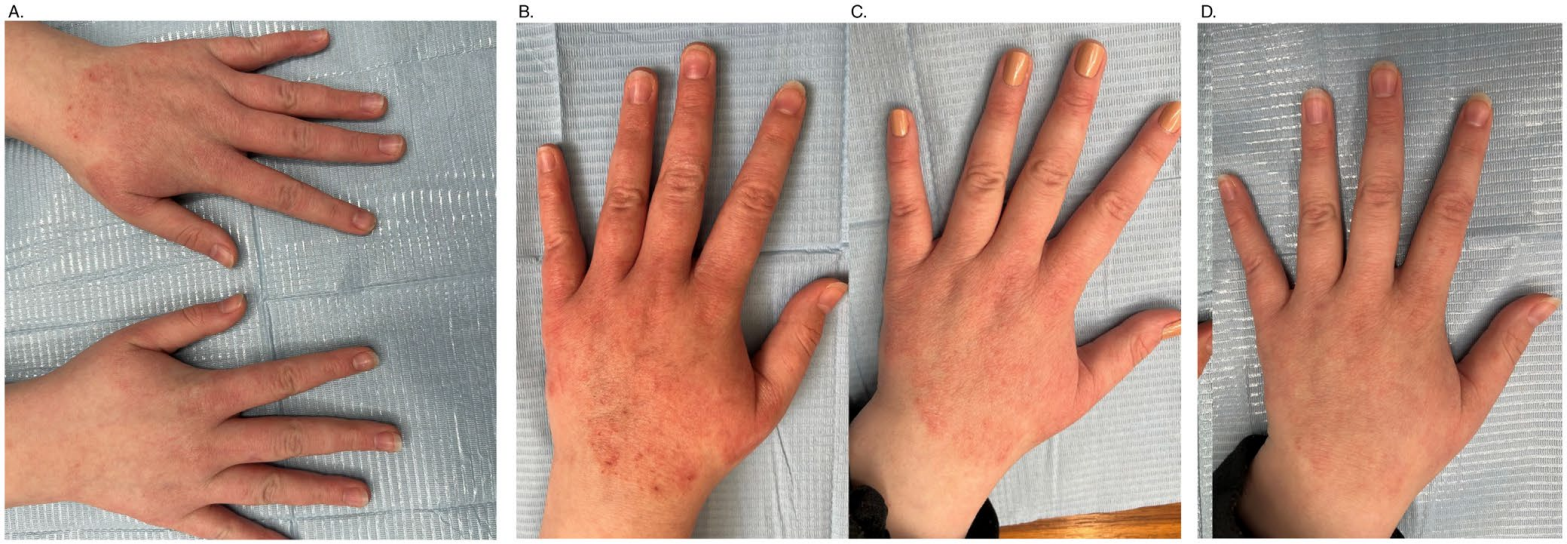
(A) Atopic dermatitis (AD)—both hands—before treatment, (B) AD—left hand—before treatment, (C) improvement of AD symptoms at Day 12 after treatment, and (D) sustained improvement at Day 20 after treatment.

The patient presented with changes on her hands, which she perceived as moderately severe, accompanied by itching. The changes were more pronounced on the left hand (Figure 1A). Left hand was treated with the product ASCEplus/SRLV (ExoCoBio), that is, exosomes from Damask rose stem cells isolated using ExoSCRT technology; right hand with the previously used emollient. Initially, two layers of ASCEplus/SRLV were applied. The rest of the product was given to the patient to apply twice daily on the affected skin of the left hand.

Subsequent visits showed significant reduction in changes, decrease in eczema, and complete cessation of itching on the hand treated with ASCEplus/SRLV, while the changes on the hand treated with emollient remained in the same condition as initially.

According to the patient, the skin on the side treated with RSCEs became smoother, more moisturized, and the itching subsided (Figure 1B–D). The product was used for 2 weeks. During the therapy, 15 mL of the product was used. The last contact with the patient was after 6 weeks—the effect was still maintained.

### 2.2 Scars

**FIGURE 2:**
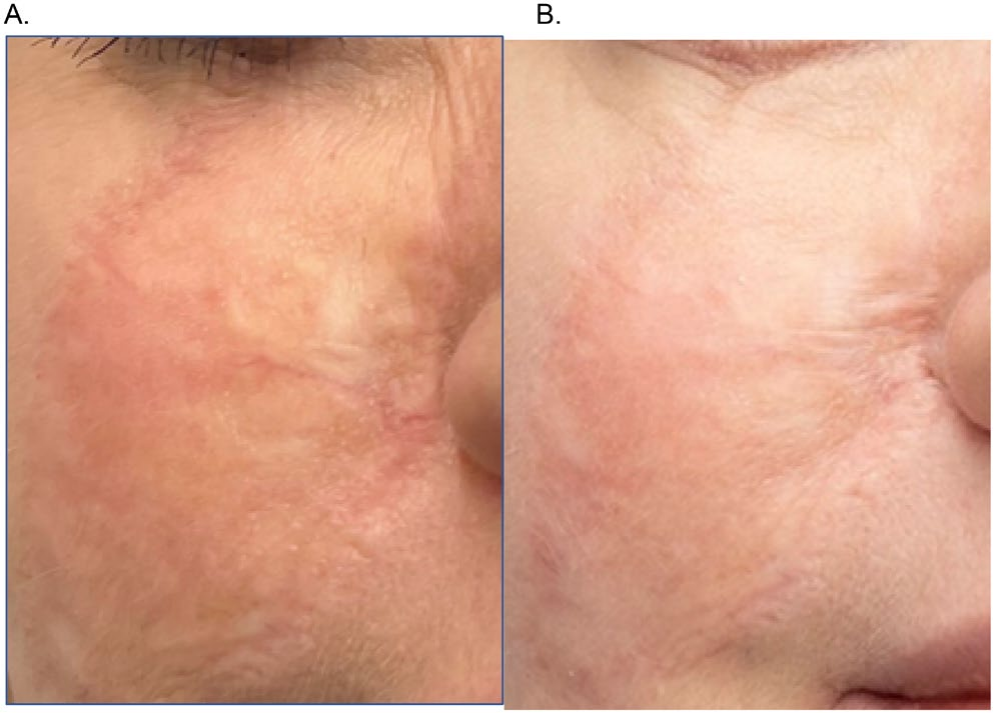
(A) Scar following autologous skin graft in the midface before treatment and (B) improvement of local conditions at Day 12 after treatment.

**FIGURE 3:**
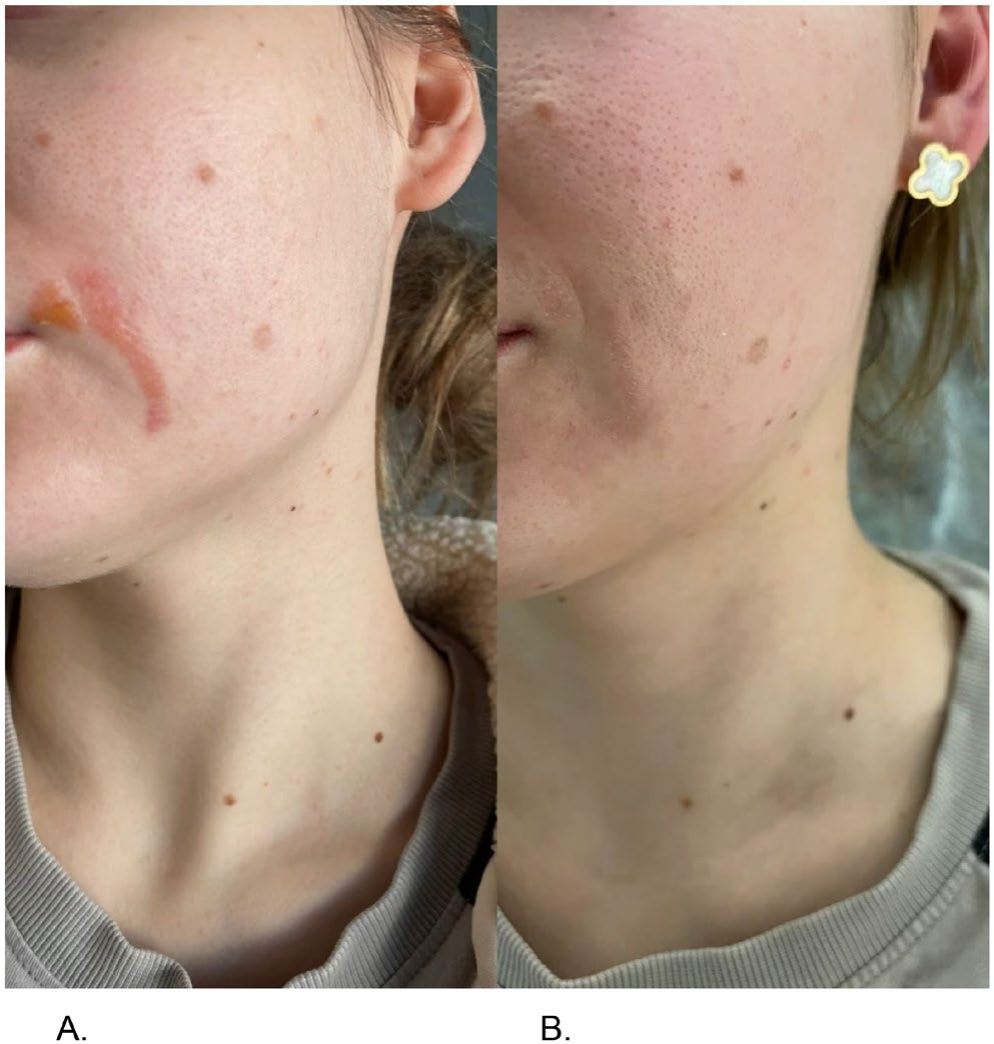
(A) Skin burn before treatment at Day 2 after the incident and (B) improvement at Day 4 after treatment.

**FIGURE 4:**
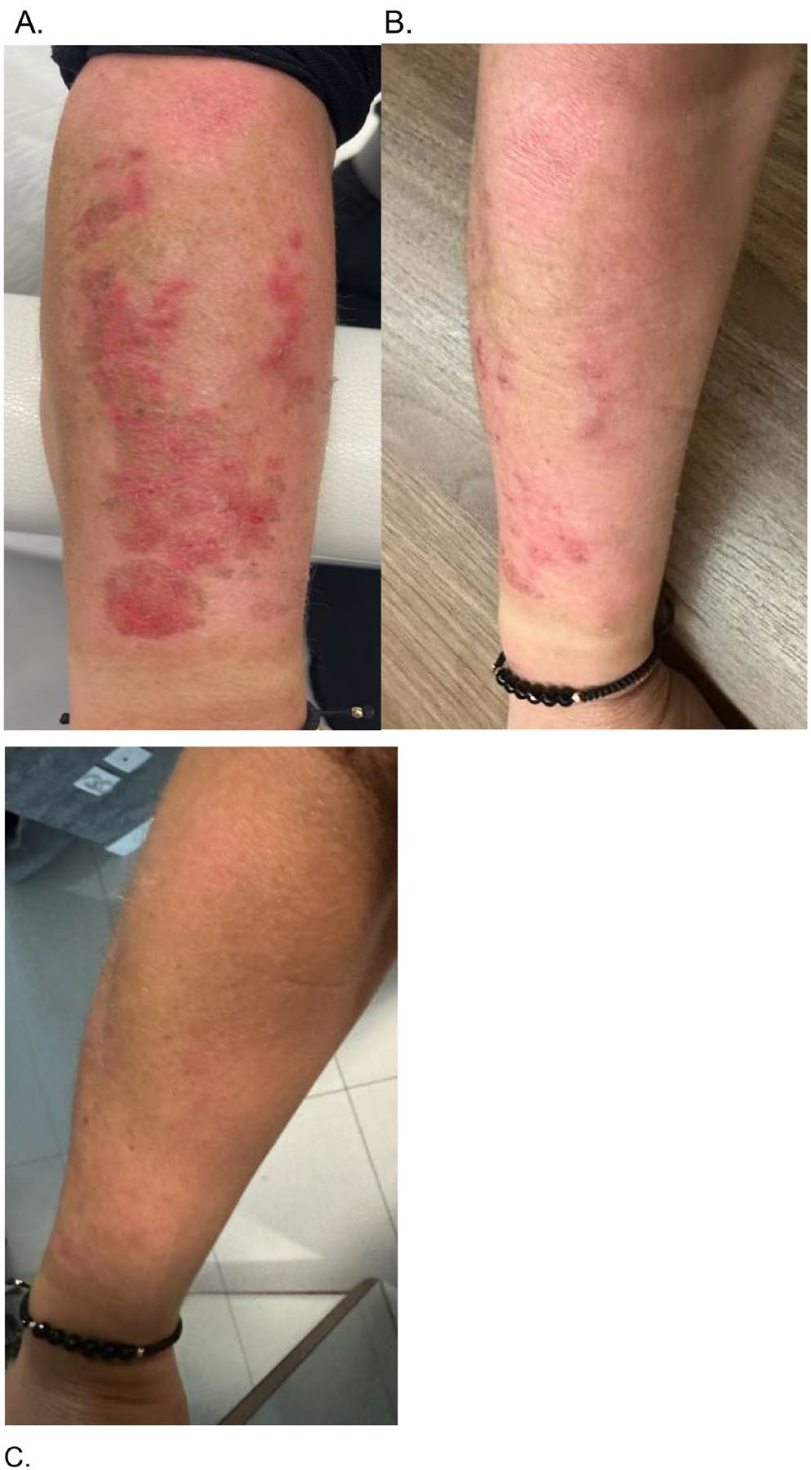
(A) Sunburn—before treatment at Day 3 post‐burn, (B) improvement of local conditions 24 h after treatment, and (C) improvement at Day 7 after treatment.

**FIGURE 5:**
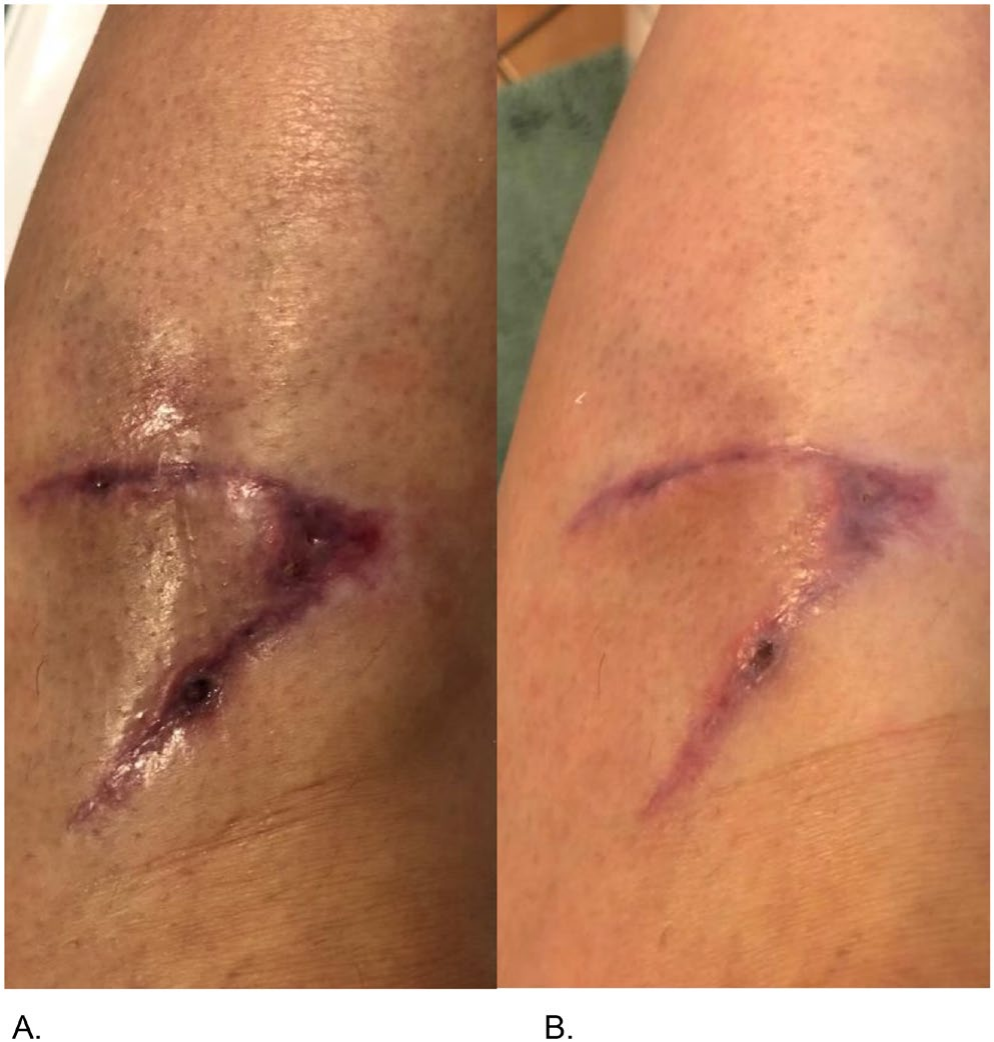
(A) Leg wound from a bicycle accident before treatment, 2 weeks after the injury and (B) improvement of the wounded site at Day 4 after treatment.

**FIGURE 6:**
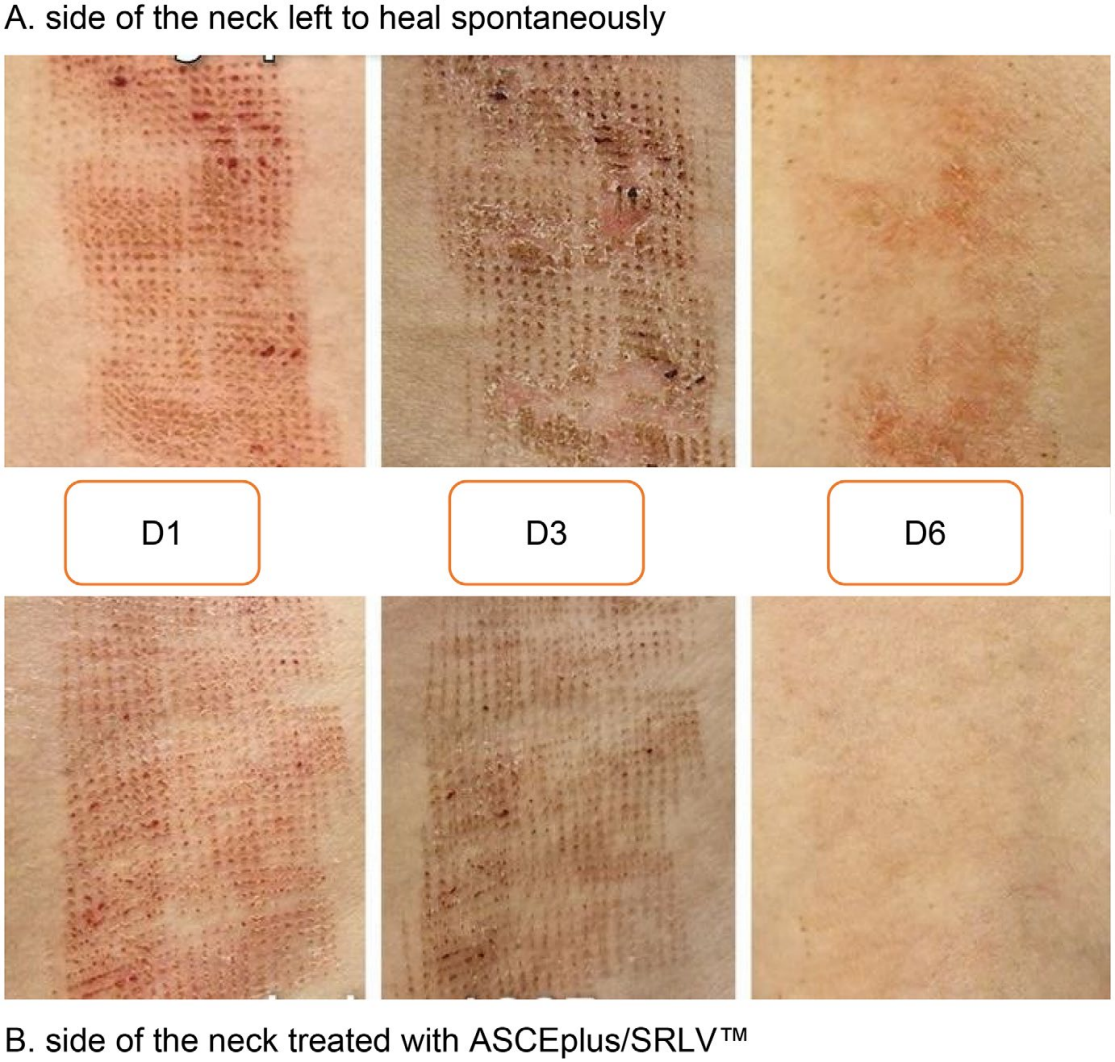
Healing after ablative fractional laser treatment, (A) side of the neck left to heal spontaneously at Day 1 (D1), at Day 3 (D3), at Day 6 (D6); (B) side of the neck treated with ASCEplus/SRLV at Day 1 (D1), at Day 3 (D3), at Day 6 (D6).

**FIGURE 7:**
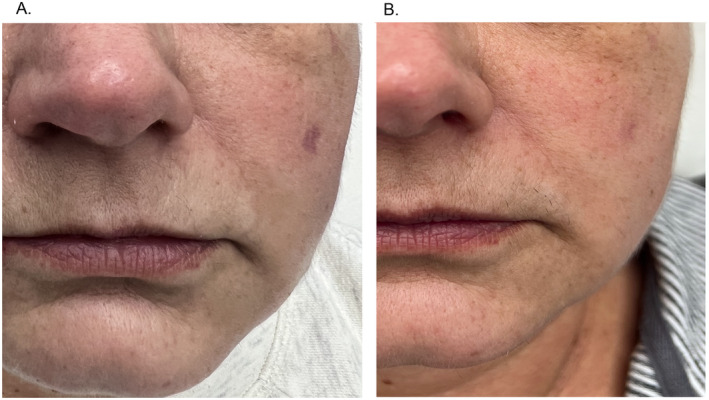
(A) Hyperpigmentation before treatment and (B) reduction of hyperpigmentation 5 weeks after treatment.

**FIGURE 8:**
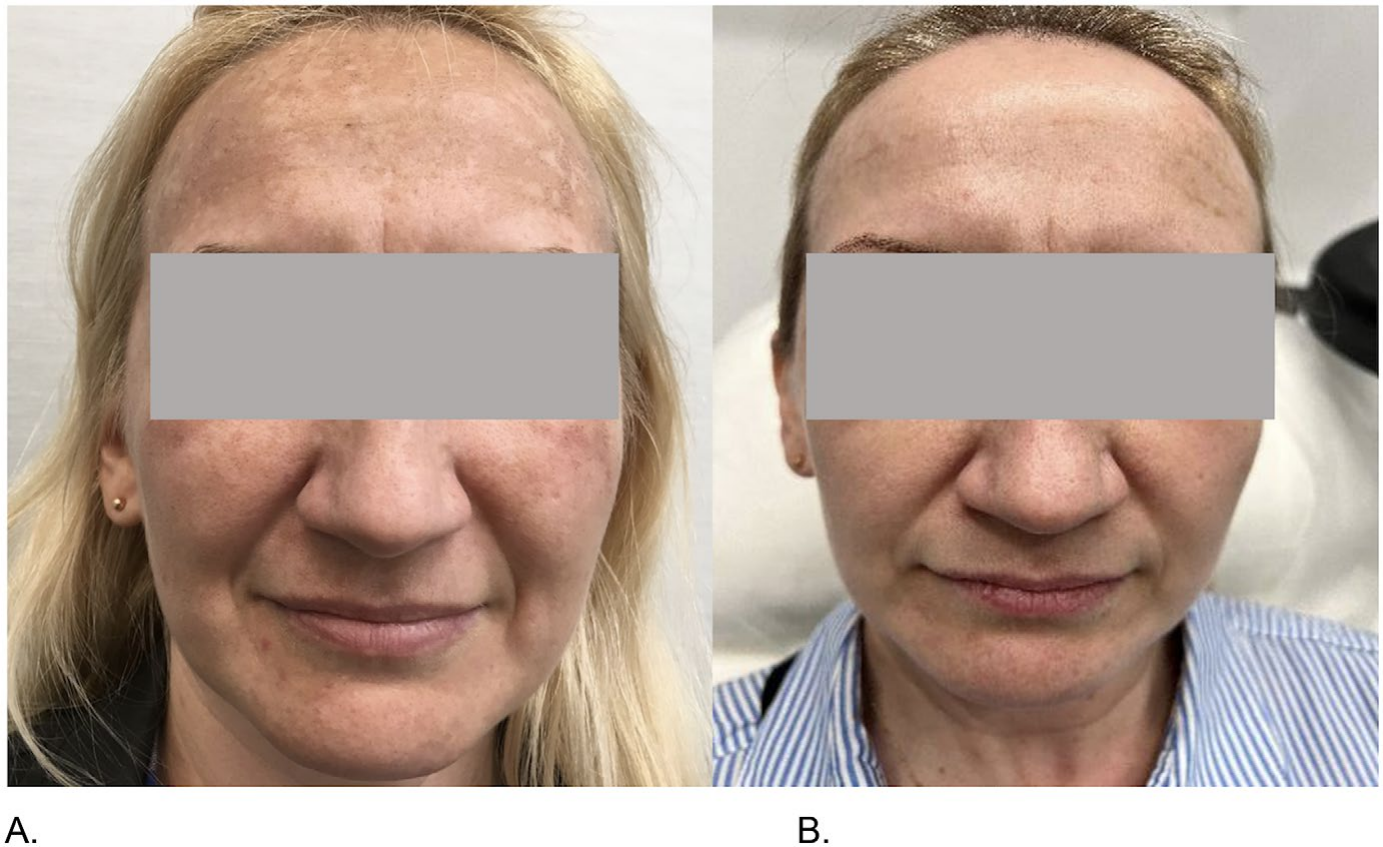
(A) Hyperpigmented facial skin before treatment and (B) reduction of hyperpigmentation 12 weeks after first treatment.

A 36‐year‐old patient had a large, pigmented nevus removed at the age of 6 from the middle part of facial skin. An autogenous skin flap from the thigh was used at the operated site. The graft integrated well, with the edges of the graft minimally distinguishable from the rest of the face. At 35 years old, the patient underwent a series of collagen injections (unknown brand) to improve the quality of facial skin. After the second treatment, she noticed thickening of the scar across the entire graft area, and the surface of the transplanted skin became irregular and thickened in some places. The attending physician decided to inject glucocorticosteroid in the graft area. Two treatments were performed at 6‐week intervals, which consequently led to skin atrophy after steroid administration, significantly worsening the appearance of that area. The patient presented about half a year after steroid administration. Microneedling treatment of the transplanted skin area to a depth of 0.5 mm (Dermapen, DermapenWorld, Australia) was performed with three needle passes over the treated surface. After the treatment, three layers of ASCEplus/SRLV were applied until the preparation was completely absorbed. The patient applied the product to the skin surface twice a day for the following days. On the seventh day, the microneedling treatment was repeated as above, continuously using RSCEs. Picture B shows the treated area on the 12th day of therapy. There is noticeable improvement of skin color, evening of skin surface sculpture, and reduced visibility of blood vessels. The patient reported that the previous pulling sensation around the nostril had completely disappeared (Figures 2, 3, 4, 5, 6, 7, 8).

### 2.3 Stimulation of the Healing Process

#### 2.3.1 Burn From a Hot Beverage

A 33‐year‐old patient, generally healthy, without any skin‐related health problems, suffered a second‐degree burn in the area of the left corner of the mouth, with blisters forming on an erythematous base. On the second day after the burn, she began applying ASCEplus/SRLV three times a day, using 10 mL of the product. No other therapy methods were included. Subsequent photographs show the healing process over the days: complete healing occurred on the sixth day after the burn.

#### 2.3.2 Sunburn

A 39‐year‐old patient, generally healthy with no skin‐related health issues (Fitzpatrick type III), suffered a second‐degree burn on the upper surface of the right forearm due to prolonged exposure to intense sun without any sun protection. The burn developed with blistering on a reddened base, which the patient peeled off on the second day due to burning sensations. On the third day post‐burn, ASCEplus/SRLV was applied twice daily to the burned skin surface. Healing of the lesions, without signs of hyperpigmentation, occurred on the seventh day of treatment (Fot. C). The entire therapy required the use of 15 mL of the product.

#### 2.3.3 Leg Wound From a Bicycle Accident

A 42‐year‐old patient, generally healthy with no skin‐related health issues, suffered a lacerated wound on the left lower leg due to a bicycle accident. The wound was disinfected, cleaned, and secured with a dressing. The patient applied a healing cream (Cicaplast Baume, La Roche Posay) to the wound surface for 2 weeks. Photograph A shows the appearance of the wound 2 weeks after the injury. At that time, ASCEplus/SRLV was started, applied twice daily to the wound surface. Photograph B shows the condition after 4 days of applying a total dose of 20 mg of lyophilized RSCEs to the skin surface.

#### 2.3.4 Healing After Ablative Fractional Laser Treatment

Ablative fractional Er‐YAG laser treatment (Pixel Er:YAG, Alma Lasers, Israel) at a dose of 1900 J, two passes, was applied to the neck skin of a 37‐year‐old man. One side of the neck was left to heal spontaneously, while RSCEs were applied twice daily to the other side. According to the patient's report, the ASCEplus/SRLV‐treated side experienced significantly less pain compared to the untreated side. Additionally, swelling, redness, and itching were markedly less on the treated side. On the sixth day after the procedure, the changes on the RSCEs‐treated side had completely healed, while the other side was still in the healing process with noticeable redness. The entire therapy required the use of 10 mL of the product.

### 2.4 Hyperpigmentations

- A 61‐year‐old patient sought treatment for visible skin discoloration on her face. The patient was generally healthy, with no skin‐related health issues, and had not used UV protection. She had not undergone any previous aesthetic medicine procedures. A microneedling procedure (Dermapen, DermapenWorld, Australia) was performed on the face surface, to a depth of 1–1.5 mm, including 1.5 mm on the cheek skin, followed by the application of ASCEplus/SRLV. Three layers of the product were applied until fully absorbed. The patient continued applying the product for 5 days, using 5 mL of the product in total. Photograph B shows the effect 5 weeks after the procedure.
- A 42‐year‐old patient presented with melasma, particularly intense on the forehead and the cheeks. A microneedling procedure (Dermapen, DermapenWorld, Australia) was performed on the face surface, to a depth of 1–1.5 mm, including 1.5 mm on the cheek and forehead skin, followed by the application of ASCEplus/SRLV. Three layers of the product were applied until fully absorbed. The patient continued applying the product for 7 days post‐procedure. Four weeks later, the microneedling procedure and ASCEplus/SRLV application were repeated. Photograph B shows the patient's face 12 weeks after the first procedure. According to the patient's report, besides the visible reduction in discoloration, the skin surface became significantly smoother and more moisturized. The entire therapy required the use of 20 mL of the product (80 mg of lyophilized RSCEs).

## 3 Discussion

The number of publications on plant‐derived EVs is growing, indicating that they are an effective alternative to human‐derived EVs. However, the number of registered clinical trials using plant EV therapy remains low. The first such studies were registered in the United States in 2012 and are still ongoing [26]. Nevertheless, in vitro, ex vivo, and animal model studies demonstrate the effectiveness of plant‐derived EVs or exosomes in treating selected diseases such as Alzheimer's disease, chronic kidney disease, stroke, non‐small‐cell lung cancer, and liver cancer [27].

Although plant‐derived exosome nanoparticles (PENs) are a relatively recent discovery, they have already been used in treatment of various intestinal diseases due to their beneficial effects and tissue‐specific targeting [28]. Specifically, reports describe their use in treating intestinal bowel disease. GELNs have been used to relieve dextran sulfate sodium (DSS)‐induced colitis in mice [19]. Another interesting therapeutic potential of PENs is the application of ginger‐derived exosome nanoparticles (GDENs) in protecting against alcohol‐induced liver damage [29]. In an animal model study, it was demonstrated that GDENs significantly reduce the symptoms of the so‐called “cytokine storm” induced during lung inflammation due to SARS‐CoV‐2 infection [30]. Another study [31] showed that ginger‐derived lipid vectors (GDLVs), which have low cytotoxicity and high biocompatibility compared to synthetic‐origin liposomes, can inhibit the uptake of iron ions in intestinal epithelial cells in cases of hereditary hemochromatosis. Another study also demonstrated the effectiveness of GELNs in reducing the inflammatory process in the intestinal epithelium [32].

Each type of PEN has different characteristics and components based on its cell of origin. Consequently, PENs and their intrinsic molecules exhibit unique regulatory patterns of signaling pathways through various mechanisms and cellular uptake processes. Similar to human‐derived exosomes, PENs also contain biomolecules such as RNAs, proteins, and lipids that regulate physiological functions. While PENs themselves can be used as transport vesicles, the structural and functional biomolecules they contain also have clinical applications [33].

The Rosa plant has various biological functions, including antioxidant, anti‐inflammatory, and anti‐microbial activities, mainly due to its content of flavonoids, polyphenols, and anthocyanins. It has been used in traditional medicine in treating various disorders, including skin‐related ones [34]. The study [35] confirms the skin anti‐inflammatory activity of rose petal extract (RPE) containing flavonoids, anthocyanins, and polyphenols and the mechanism underlying this effect. The researchers found that RPE reduces solar UV‐induced expression of COX‐2 and causes inhibition of several cytokines. Additionally, RPEs strong antioxidant activity, which contributes to its anti‐inflammatory effects, was demonstrated. The authors conclude: “while the bioactive chemical in RPE is yet to be identified, the present study suggests that 70% ethanol extract from rose petals exhibit skin anti‐inflammatory and antioxidant activities via MAPK inactivation.” Findings of another study [36] suggest that RPE and its active component cyclic digalactosyl‐diacylglycerol (CDG) increase skin hydration by upregulating HAS2 expression. The results of a recently published study [25] show the effects of RSCEs on cell functions relevant to skin. These functions include growth of skin fibroblasts and collagen production, reduced melanin production in melanocytes, and inhibition of inflammation. This study used cryo‐electron microscopy to demonstrate the presence of round vesicles with a diameter of about 100–200 nm and other components, including RNA. To determine whether RSCEs can affect melanin production, the mouse melanoma cell line was used. Literature has already shown the effect of human adipose‐derived stem cell exosomes on skin lightening [37], but there is still no research on the effect of plant‐derived exosomes in this indication.

The efficacy of exosomes derived from Damask Rose stem cells (RSCEs) was demonstrated in a recently published study that combined RSCE therapy with microneedling [38]. The study, conducted on a group of 20 individuals with melasma, showed a reduction in the modified Melasma Area Severity Index (mMASI) after five sessions of microneedling with the application of RSCEs. A significant improvement in the mMASI score was observed in 90% of the participants, with 40% showing improvement to mild mMASI and 60% to moderate mMASI. The authors emphasized that no serious adverse events or post‐inflammatory hyperpigmentation were observed in individuals with darker skin tones (Fitzpatrick III), indicating a high safety profile of the applied therapy.

It was demonstrated [25] that RSCEs are taken up by melanocytes and affect the reduction of melanin production, suggesting a skin‐lightening effect. The earlier presented examples of patients with hyperpigmentation and the effects of therapy using RSCEs support this thesis. However, the molecular mechanism of this action is not clear. The study [25] also showed that the level of melanin reduction in melanocytes is dose‐dependent. The most significant effect is observed 48 h after application, achieving a result comparable to the positive control using arbutin.

The effect of exosomes derived from human adipose‐derived stem cells (ADSCs‐Exos) on wound healing is described in numerous studies [11, 39, 40, 41]. This process is complex depending on the tissue healing phase. In the wound phase, the action of ADSCs‐Exos increased the migration and proliferation of fibroblasts and the expression of collagen Type I, collagen Type II, decorin, elastin, and matrix metalloproteinase 3. In the scar formation phase, however, exosomes derived from human adipose‐derived stem cells decreased fibroblast migration, proliferation, and differentiation, as well as the expression of collagen Type I, collagen Type III, beta‐smooth muscle actin, phosphorylated p38 mitogen‐activated protein kinase, and tissue inhibitor of metalloproteinases. The levels of alpha‐smooth muscle actin and transforming growth factor beta 1 remained reduced in both phases, preventing the transformation of fibroblasts to myofibroblasts. The mechanism of action of plant‐derived exosomes on scars is not described. In the study [25], an in vitro scratch assay was used, which simulates the action of fibroblasts in wound healing. In this model, it was shown that RSCEs caused wound closure, likely due to stimulation of fibroblast proliferation and migration, but also due to intracellular delivery of biomolecules, including RNA.

The above examples of healing wounds of various origins and remodeling scarred tissue seem to confirm the effectiveness of RSCEs in these applications.

Due to numerous studies and the steadily growing number of publications on the applications and clinical effects of PENs, exosomes from plant stem cells are now becoming accepted as potential next‐generation cell‐free therapeutic agents. The undeniable advantages include the high availability of plant sources, the ability to produce large quantities of PENs in a short time, and their biocompatibility. However, there are still many challenges associated with the commercialization of exosome‐containing products, such as large‐scale stem cell cultivation, continuous supply of products with comparable therapeutic effects, and precise quantification and quality assessment of exosomes. Nevertheless, technological advancements in PENs‐based therapies and cell engineering provide hope for the widespread market approval of such products, thus introducing effective therapies for many diseases.

## 4 Conclusion

In this case series, we describe the results of using RSCEs to improve the skin quality of patients with AD, wounds of various origins, and burns. RSCEs contribute to the treatment of these conditions by inhibition of the inflammatory process and hyperpigmentation, based on our own observations. We emphasize that these results were obtained using only plant‐derived exosomes, from the stem cells of the Damask rose. Given that, apart from the earlier cited work [25], there is no available literature on this topic, this work is an important contribution to the general knowledge about the action of plant‐derived exosomes on skin functions. We are aware, however, that there is an urgent need for multicenter randomized and controlled clinical trials to confirm the theses obtained based on practical observations.

## Author Contributions

Conceptualization: All authors have read and agreed to the published version of the manuscript. All authors gave their final approval and agreed to be accountable for all aspects of the work.

## Ethics Statement

The authors declare that all procedures were performed in adherence to the Declaration of Helsinki, in accordance with regional laws and good clinical practice. All patients have signed a consent form for the procedure and for the publication of their images.

## Conflicts of Interest

The authors declare no conflicts of interest.

## Supporting information


File S1.

## Data Availability

The data that support the findings of this study are available from the corresponding author upon reasonable request.
