# Supplementary material for: Efficacy of Rose Stem Cell‐Derived Exosomes (RSCEs) in Skin Treatment: From Healing to Hyperpigmentation Management: Case Series and Review
Source: J Cosmet Dermatol. 2025 Jan 15;24(1):e16776. doi: 10.1111/jocd.16776 (PMC11736088; doi:10.1111/jocd.16776)
Supplement: Supplementary file 1 — File S1. [file JOCD-24-e16776-s001.pdf]

ExoCoBio Inc.

#306, 19, Gasan digital 1-ro, Geumcheon-gu, Seoul, Korea
TEL : +82-2-2038-3915, FAX : +82-2-2038-3916

[FORMULAR INGREDIENTS STATEMENT]

PRODUCT NAME : ASCE^{plus} Derma Signal Kit SRLV [Vial 1 : Lyophilized Powder]

No.	INCI Name	Percentage	CAS No.	Function
1	Rosa Damascena Callus Extracellular Vesicles	40.00	-	Skin Conditioning Agent
2	Methionine	2.00	63-68-3, 59-51-8	Skin Conditioning Agent
3	Trehalose	10.00	6138-23-4, 99-20-7	Humectant
4	Mannitol	10.00	87-78-5, 69-65-8	Binder
5	Glutamine	1.00	56-85-9	Skin Conditioning Agent
6	Potassium Chloride	1.00	7447-40-7	Viscosity Increasing Agent
7	Ascorbic Acid	1.00	50-81-7	Skin Conditioning Agent
8	Retinol	1.00	68-26-8, 11103-57-4	Skin Conditioning Agent
9	Magnesium Sulfate	1.00	10034-99-8	Bulking agents
10	Glutathione	1.00	70-18-8	Reducing agent
11	Nicotinamide Adenine Dinucleotide	1.00	53-84-9	Skin Conditioning Agent
12	Disodium Flavine Adenine Dinucleotide	1.00	146-14-5	Skin Conditioning Agent
13	Thiamine Diphosphate	1.00	154-87-0	Skin Conditioning Agent
14	Coenzyme A	1.00	85-61-0	Skin Conditioning Agent
15	sh-Oligopeptide-2	1.00	-	Skin Conditioning Agent
16	Acetyl Hexapeptide-8	1.00	616204-22-9	Skin Conditioning Agent
17	Nonapeptide-1	1.00	-	Skin Conditioning Agent
18	Palmitoyl Tetrapeptide-7	1.00	221227-05-0	Skin Conditioning Agent
19	Palmitoyl Tripeptide-1	1.00	-	Skin Conditioning Agent
20	sh-Oligopeptide-1	1.00	-	Skin Conditioning Agent
21	sh-Polypeptide-1	1.00	-	Skin Conditioning Agent
22	sh-Polypeptide-3	1.00	-	Skin Conditioning Agent
23	sh-Polypeptide-76	1.00	-	Skin Conditioning Agent
24	Copper Tripeptide-1	1.00	-	Skin Conditioning Agent
25	Palmitoyl Pentapeptide-4	1.00	214047-00-4, 521091-64-5	Skin Conditioning Agent
26	Alanine	1.00	56-41-7	Skin Conditioning Agent
27	Arginine	1.00	74-79-3	Skin Conditioning Agent
28	Aspartic Acid	1.00	56-84-8	Skin Conditioning Agent
29	Glutamic acid	1.00	56-86-0	Skin Conditioning Agent
30	Glycine	1.00	56-40-6	Skin Conditioning Agent
31	Histidine	1.00	71-00-1	Skin Conditioning Agent
32	Isoleucine	1.00	73-32-5	Skin Conditioning Agent

33	Leucine	1.00	61-90-5	Skin Conditioning Agent
34	Lysine HCL	1.00	10098-89-2	Skin Conditioning Agent
35	Ornithine HCL	1.00	3184-13-2	Skin Conditioning Agent
36	Phenylalanine	1.00	63-91-2, 6<br>2056-68-2	Skin Conditioning Agent
37	Proline	1.00	147-85-3	Skin Conditioning Agent
38	Serine	1.00	56-45-1	Skin Conditioning Agent
39	Threonine	1.00	72-19-5	Skin Conditioning Agent
40	Tryptophan	1.00	73-22-3	Skin Conditioning Agent
41	Tyrosine	1.00	60-18-4	Skin Conditioning Agent
42	Valine	1.00	72-18-4	Skin Conditioning Agent
Total		100.00		

ExoCoBio Inc.

#306, 19, Gasan digital 1-ro, Geumcheon-gu, Seoul, Korea

TEL : +82-2-2038-3915, FAX : +82-2-2038-3916

[FORMULAR INGREDIENTS STATEMENT]

PRODUCT NAME : ASCE^{plus} Derma Signal Kit SRLV [Vial 2 : Diluent]

No.	INCI Name	Percentage	CAS No.	Function
1	Water	97.70	7732-18-5	Solvent
2	Sodium Hyaluronate	0.70	9067-32-7	Skin Conditioning Agent
3	Sodium Chloride	0.50	7647-14-5	Viscosity Increasing Agent
4	Sodium Bicarbonate	0.15	144-55-8	ph Adjusters
5	Disodium Phosphate	0.10	10028-24-7	ph Adjusters
6	Sodium Phosphate	0.05	13472-35-0	ph Adjusters
7	Potassium Chloride	0.05	7447-40-7	Viscosity Increasing Agent
8	Alanine	0.05	56-41-7	Skin Conditioning Agent
9	Arginine	0.05	74-79-3	Skin Conditioning Agent
10	Histidine	0.05	71-00-1	Skin Conditioning Agent
11	Isoleucine	0.05	73-32-5	Skin Conditioning Agent
12	Leucine	0.05	61-90-5	Skin Conditioning Agent
13	Lysine HCL	0.05	10098-89-2	Skin Conditioning Agent
14	Phenylalanine	0.05	63-91-2, 62056-68-2	Skin Conditioning Agent
15	Proline	0.05	147-85-3	Skin Conditioning Agent
16	Serine	0.05	56-45-1	Skin Conditioning Agent
17	Threonine	0.05	72-19-5	Skin Conditioning Agent
18	Valine	0.05	72-18-4	Skin Conditioning Agent
19	sh-Decapeptide-7	0.05	-	Skin Protector
20	sh-Octapeptide-4	0.05	-	Skin Protector
21	sh-Oligopeptide-9	0.05	-	Skin Conditioning Agent
22	sh-Pentapeptide-5	0.05	-	Skin Conditioning Agent
Total		100.00		
